# Matrix Metalloproteinase Gene Polymorphisms and Bronchopulmonary Dysplasia: Identification of MMP16 as a New Player in Lung Development

**DOI:** 10.1371/journal.pone.0003188

**Published:** 2008-09-11

**Authors:** Alice Hadchouel, Fabrice Decobert, Marie-Laure Franco-Montoya, Isabelle Halphen, Pierre-Henri Jarreau, Olivier Boucherat, Emmanuel Martin, Alexandra Benachi, Serge Amselem, Jacques Bourbon, Claude Danan, Christophe Delacourt

**Affiliations:** 1 INSERM, Unité 841, IMRB, équipe 06, Créteil, France; 2 PremUp, Paris, France; 3 Réanimation Néonatale, Centre Hospitalier Intercommunal, Créteil, France; 4 Service de médecine néonatale de Port-Royal, AP-HP, Hôpital Cochin, Paris, France; 5 IntegraGen, Evry, France; 6 Service d'Obstétrique et Gynécologie, AP-HP, Hôpital Necker, Paris, France; 7 INSERM U654, GH Armand Trousseau, Paris, France; 8 Unité Fonctionnelle de Recherche Clinique, Centre Hospitalier Intercommunal, Créteil, France; 9 Université Paris 12, Faculté de Médecine, IFR10, Créteil, France; 10 Université Paris Descartes, Faculté de Médecine, Paris, France; University of Giessen Lung Center, Germany

## Abstract

**Backgound:**

Alveolarization requires coordinated extracellular matrix remodeling, a process in which matrix metalloproteinases (MMPs) play an important role. We postulated that polymorphisms in MMP genes might affect MMP function in preterm lungs and thus influence the risk of bronchopulmonary dysplasia (BPD).

**Methods and Findings:**

Two hundred and eighty-four consecutive neonates with a gestational age of <28 weeks were included in this prospective study. Forty-five neonates developed BPD. Nine single-nucleotide polymorphisms (SNPs) were sought in the MMP2, MMP14 and MMP16 genes. After adjustment for birth weight and ethnic origin, the TT genotype of MMP16 C/T (rs2664352) and the GG genotype of MMP16 A/G (rs2664349) were found to protect from BPD. These genotypes were also associated with a smaller active fraction of MMP2 and with a 3-fold-lower MMP16 protein level in tracheal aspirates collected within 3 days after birth. Further evaluation of MMP16 expression during the course of normal human and rat lung development showed relatively low expression during the canalicular and saccular stages and a clear increase in both mRNA and protein levels during the alveolar stage. In two newborn rat models of arrested alveolarization the lung MMP16 mRNA level was less than 50% of normal.

**Conclusions:**

MMP16 may be involved in the development of lung alveoli. MMP16 polymorphisms appear to influence not only the pulmonary expression and function of MMP16 but also the risk of BPD in premature infants.

## Introduction

Despite major advances in the care of very-low-birth-weight (VLBW) infants, bronchopulmonary dysplasia (BPD) still affects 20 to 40% of survivors [1]. BPD appears to result from arrested lung development and is characterized by abnormal alveolar septation and abnormal microvascular maturation [2]. Alveolarization requires coordination of extracellular matrix remodeling with epithelial morphogenesis and capillary growth [3]. This process involves matrix metalloproteinases (MMPs), which are classified into two major groups according to their subcellular localization: membrane-type MMPs (MT-MMPs) and secreted MMPs. Among secreted MMPs, MMP2 is known to play a key role in lung development and repair after injury. Mice lacking this proteinase show delayed alveolar development [4], and low MMP2 levels in tracheal effluent and plasma have been linked to an increased risk of BPD in infants [5], [6]. Type I transmembrane MT-MMPs participate in the activation of the zymogen form of MMP2 (pro-MMP2). Among these, MT1-MMP (MMP14) has a major role in alveolarization. We recently observed a fourfold increase in MMP14 transcripts in lung fibroblasts during alveolarization [7], while mice lacking this enzyme have a reduced alveolar surface area and enlarged air spaces [8], [9], although this seems partly independent of the ability of MMP14 to activate MMP2 [9]. MT3-MMP (MMP16) was originally cloned from a human placental cDNA library [10]. Although its role in lung development has not previously been studied, its ability to localize on the cell membrane and to activate pro-MMP2 has been clearly demonstrated. MMP16 is also known to have a splice variant, consisting of a soluble form lacking the transmembrane domain. This soluble form can also activate pro-MMP2 [11].

Although exposure of the immature lung to various insults is thought to play a central role in the onset of BPD, significant genetic susceptibility to BPD was recently demonstrated in preterm infants [12]. Hereditary differences in the expression of genes critical for lung development may therefore have a role in BPD pathogenesis. We postulated that polymorphisms in MMP genes might affect MMP function in preterm lung and thereby influence the risk of BPD at a given gestational age. Indeed, a single-nucleotide polymorphism (SNP) in the promoter of the MMP2 gene (−1306 C/T) modulates the promoter activity of MMP2 and has functional significance [13]. In addition, a haplotype of four MMP14 polymorphisms (−130 T: +256 T: +6762 C: +7131 C) has been linked to chronic obstructive pulmonary disease [14]. Finally, the MMP16 splice variant might potentially be generated by polymorphism of the gene region coding for the hemopexin domain [11].

Our initial aim in this study was to examine whether any of nine SNPs in the MMP2, MMP14, and MMP16 genes was associated with BPD in premature infants, and with the level of MMP2 activity in tracheal effluents. As we found a significant association with two SNPs in the MMP16 gene, we further examined the expression of this enzyme during normal human and rat lung development, and in newborn rat models of arrested alveolar development.

## Methods

### Study population

Two hundred and eighty-four neonates with a gestational age of <28 weeks were included from two neonatal intensive care units (Creteil and Cochin-Port Royal) between March 2002 and December 2006. All were inborn, and received prophylactic surfactant treatment immediately after birth, in the delivery room. The mean term was 26.4±0.1 (SE) weeks (range 23.6–27.9 weeks), and the mean birth weight was 838±11 g (440–1410 g). Perinatal factors susceptible to influence the risk for BPD were collected: bacteriologically proven maternofetal infection ; antenatal steroid therapy ; histologically defined chorioamnionitis ; persistent ductus arteriosus requiring medical or surgical treatment ; bacteriologically proven postnatal sepsis ; air leaks. The duration of ventilation support was not analyzed because of evolving strategies over study time. The diagnosis of BPD was based on the continued need for oxygen supplementation at a postmenstrual age (PMA) of 36 weeks, according to the standardized physiologic test validated by Walsh [15]. The study was approved by the local ethics committee (CCPPRB Henri Mondor), and written informed consent was obtained from the parents.

### Collection of tracheal secretions

A tracheal aspirate was obtained from 148 of the neonates immediately after birth (Day 0), before surfactant instillation. One hundred and five neonates were resampled before extubation, or on Day 3 when still intubated (mean: 2.5±0.1 postnatal days). Tracheal aspiration and sample preparation were performed as previously described [5], [16]. Briefly, suction was performed with a small catheter and was preceded by instillation of 0.2 ml of isotonic saline. The aspirate was collected in an infant mucus extractor (Vygon, Ecouen, France), then diluted in 0.2 ml of isotonic saline, gently vortexed, and centrifuged at 1200 rpm for 10 min at 4°C. The supernatant was recovered and stored at −80°C. The dilution factor induced by this technique is low [5], [16], allowing the results to be expressed per milliliter of supernatant.

### Human fetal lung tissue sampling

Lung tissue samples were obtained at autopsy after medical termination of pregnancy or neonatal death, with the parents written informed consent. The terminations complied with French legislation, and the study was approved by our institutional ethics committee. The fetuses were free of pulmonary diseases and the samples were histologically normal, with no hypoplasia. Detailed clinical data on the lung tissue donors have been reported elsewhere [17]. Fetal age (postconception) is used throughout the paper. The lung tissues were homogenized in RIPA buffer containing protease inhibitors (Roche Diagnostics, http://www.roche.com).

### Animal models and rat lung tissue

Dated pregnant Sprague-Dawley rats were purchased from Charles River (Saint Germain sur l'Arbresle, France). The day of mating was designated day 0 of gestation. Term is 22 days. Lung tissues were collected between fetal day 18 and postnatal day 21. Lung tissue from adult rats (8 weeks of age) were also collected. Fetuses were retrieved by cesarean section under pentobarbital anesthesia. Lungs from fetuses and pups were immediately frozen in liquid nitrogen and kept at −80°C until RNA extraction or immunoblot analysis. Animal experiments complied with the Guide for Care and Use of Laboratory Animals, and were authorized by the French Ministry of Agriculture.

### In vivo treatment of rat pups

We used two treatments known to induce alveolar growth disorders in newborn rats, namely hyperoxia and dexamethasone. We have previously reported resulting morphometric alterations [18].

#### Hyperoxia

Rat pups and their dams were placed in Plexiglas exposure chambers (Charles River) and run in parallel with either >95% or 21% (room air) FiO_2_ from day 0 to day 7. The oxygen concentration was regularly monitored. Because adult rats have limited resistance to high O_2_, the dams were switched daily between O_2_-exposed and room air-exposed litters. Pups from two different litters were mixed so that there were littermates in both conditions. The chambers were opened for 20 min every day to switch dams between the air and O_2_ environments and to clean the cages. On day 7, the pups were killed by intraperitoneal sodium pentobarbital injection (70 mg/kg, Ceva, Libourne, France), and exsanguinated by aortic transsection. The lungs were immediately placed in liquid nitrogen and kept at −80°C until RNA or protein extraction.

#### Dexamethasone (Dex) treatment

Two different litters were subdivided into three groups of animals, that received an intraperitoneal injection of 0.1 or 0.5 µg/g/day water-soluble Dex (Sigma, L'Isle d'Abeau, France), or the vehicle alone (saline, control group), from birth to day 5. Five animals were used for each experimental condition. The pups were killed on day 6 as described above.

### MMP2 measurements in tracheal aspirates

A selective ELISA system (R&D Systems, Abingdon, UK) was used to determine total MMP2 concentrations in tracheal aspirate supernatants, according to the manufacturer's instructions. MMP2 activity was evaluated by zymography, as previously described [5]. Supernatants of tracheal aspirates were analyzed by electrophoresis in 8% (wt/vol) polyacrylamide gels containing 1 mg/ml gelatin in the presence of SDS in nonreducing conditions. After electrophoresis, the gels were washed in 2.5% Triton X-100 for 1 h and then rinsed briefly and incubated at 37°C for 24 h in buffer containing 100 mM Tris-HCl, pH 7.40, and 10 mM CaCl2. The gels were then stained with Coomassie brillant blue R250 and restained in a solution of 7.5% acetic acid and 5% methanol. Enzyme activities in the gel slabs were quantified by means of image analysis (NIH Image software 1.52 for Macintosh), on the basis of both the surface area and the intensity of lysis bands. Results were expressed as arbitrary units (AU) per 24 h per microliter of supernatant. The total MMP2 level was calculated as the sum of activities measured at 72 kDa (proenzyme) and 68 kDa (activated enzyme). The fraction of activated gelatinase was calculated as the activated/total enzyme ratio.

### Immunoblot analysis

Immunoblot analysis was applied to supernatants of tracheal aspirates and to human and rat lung tissue homogenates. Protein content was assayed in each sample with the Bradford assay. A fixed amount of total protein (12 µg, 80 µg, and 50 µg for tracheal aspirates, human lung tissue, and rat lung tissue, respectively) was electrophoresed on 8 to 10% SDS-polyacrylamide gels then transferred to polyvinylidene-fluoride membranes (Millipore, Saint-Quentin en Yvelines, France), then stained with Ponceau S dye (Sigma, http://www.sigmaaldrich.com). After blocking with 5% nonfat dry milk in Tris-buffer saline containing 0.1% Tween-20 (TTBS) at room temperature for 90 min, the membranes were exposed to a rabbit polyclonal antibody against MMP16 (Abcam, Cambridge, UK), diluted 1∶1000 in 2% non fat dry milk in TTBS at room temperature for 2 h. After five rinses in TTBS, the membranes were incubated for 1 h with a secondary peroxidase-conjugated IgG antibody (Dako, Trappes, France) diluted 1∶2500. The membranes were then incubated for 1 min in chemiluminescent detection reagent (ECL, GE Healthcare Life Sciences, Velizy, France) before exposure to KODAK BioMax MS film for 15 min. NIH image software was used for densitometric analysis of the blots.

### Genotyping

Genomic DNA was extracted from cord-blood leukocytes by using the Nucleon kit (Amersham). The SNPs studied here are summarized Table 1. SNPs in the MMP2 and MMP14 genes that had previously been linked to respiratory diseases were included in this study, namely −1306 C/T in the MMP2 gene [19], [20], and −129 G/T, +256 T/C, +6762 C/G, and +7131 T/C in the MMP14 gene [14]. +6802 G/A and +6926 C/T SNPs were also included, based on their exonic location. Two “tag” SNPs (rs2664349 and rs2664352) that determine a haplotype in the region of the MMP16 gene encoding the hemopexin domain were selected from HapMap data.

**Table 1:** Single-nucleotide polymorphisms (SNPs) studied in the MMP2, MMP14, and MMP16 genes.

Gene	Genomic location	Reference	Alleles	Fluorophores*
MMP2	−1306	rs243865	C/T	-
MMP14	−129	rs1003349	G/T	G: VIC
				T: FAM
MMP14	+256	rs1042703	T/C	T: FAM
				C: VIC
MMP14	+6762	rs2236302	C/G	-
MMP14	+6802	rs1042704	G/A	-
MMP14	+6926	rs2236303	C/T	-
MMP14	+7131	rs2236307	T/C	-
MMP16	+39811	rs2664349	A/G	A: VIC
				G: FAM
MMP16	+43827	rs2664352	C/T	C: VIC
				T: FAM

* Allele-specific fluorogenic probes used for genotyping with the TaqMan® technique.

The −1306 C/T genotype was determined by polymerase chain reaction amplification (PCR) followed by restriction fragment length polymorphism analysis (RFLP). The forward and reverse primers were designed with Primer Express software (Applied Biosystems), as follows: 5′-CTT CCT AGG CTG GTC CTT ACT GA-3′ and 5′-CTG AGA CCT GAA GAG CTA AAG AG**C** T-3′. The penultimate nucleotide of the reverse primer was modified (G replaced by **C**) in order to create a restriction site for Bfa1. PCR was carried out in a 50-µL volume containing 200 ng of genomic DNA, 5 µL of 10× PCR Rxn Buffer (Invitrogen), 2 µL of 50 mM MgCl_2_ (Invitrogen), 1 µL of nucleotide mix (dNTP 10 mM, Invitrogen), 13 pmol of forward and reverse primer and 1.5 units of Taq polymerase (Recombinant Taq DNA Polymerase, Invitrogen). The thermal cycling conditions were 94°C for 5 min, 37 cycles of 94°C for 1 min, 59°C for 1 min and 72°C for 1 min, then 72°C for 10 min. Enzymatic digestion was carried out in a 20-µL volume containing 3 µL of PCR product, 7.5 units of Bfa1 (New England Biolabs) and 2 µL of 1× Nebbuffer 4 (New England Biolabs). This mix was then incubated at 37°C for 4 hours. The size and number of the different fragments were determined by electrophoretic migration on ethidium bromide-stained 3% agarose gel. We expected to obtain one 188-bp fragment for the CC genotype, two fragments (162 and 26 bp) for the TT genotype, and three fragments (188, 162 and 26 bp) for the CT genotype.

The +6762 C/G, +6802 G/A, +6926 C/T and +7131 T/C genotypes were determined by PCR followed by DNA sequencing. The forward and reverse primers were designed using Primer Express software (Applied Biosystems), as follows: 5′-GAGGCTGAGGGAAGGGACTC-3′ and 5′-GGGTTTTTGGGTTTATCAGGAAC-3′, producing a 590-bp fragment containing the four SNPs. The PCR mix and thermal cycling conditions were the same as described above for −1306 C/T. Sequencing was carried out in a 10-µL volume containing 2 to 5 µL of PCR product, 2 µL of sequencing mix (Big Dye Terminator 3.1) and 10 pmol of forward and reverse primer. The thermal cycling conditions were 96°C for 2 min, then 25 cycles of 96°C for 10 sec, 55°C for 5 sec and 60°C for 1 min. Sequencing was performed by the Molecular Medicine Unit of Henri Mondor Hospital (IM3). Sequences were read in our laboratory with Chromas Pro 1.34 software.

Genotype analysis of −129 G/T, +256 T/C, rs2664349 A/G and rs2664352 C/T was performed with allele-specific fluorogenic probes and the TaqMan® technique. Primers and probes were designed and synthesized by Applied Biosystems and provided as an SNP Genotyping Assay Mix specific for each SNP. Probes were labeled with the fluorophores, 6-carbofluorescein (FAM) or VIC (Table 1), and were minor groove binder (MGB) probes. PCR was carried out in a 20-µL volume containing 10 ng of genomic DNA, 10 µL of TaqMan® Genotyping Master Mix (Applied Biosystems) and 1 µL of TaqMan® SNP Genotyping Assay Mix 20× (Applied Biosystems). Thermal cycling conditions were 95°C for 10 min, then 40 cycles of 92°C for 15 sec and 60°C for 1 min in the ABI PRISM 7000 (Applied Biosystems). The genotypes were determined by reading the fluorescent signal of FAM and VIC from the end-products.

### Real-time quantitative PCR (qPCR)

RNA from each sample extract was reverse-transcribed into cDNA by using 2 µg of total RNA, Superscript II reverse transcriptase, and random hexamer primers (Invitrogen) according to the supplier's protocol. Realtime PCR was performed on an ABI Prism 7000 (Applied Biosystems, Courtaboeuf, France) using the following protocol: initial denaturation (10 min at 95°C), then two-step amplification program (15 s at 95°C followed by 1 min at 60°C) repeated 40 times. Melt curve analysis was used to check that a single specific amplified product was generated. Reaction mixtures consisted of 25 ng cDNA, SYBR Green 2× PCR Master Mix (Applied Biosystems), and forward (5′-GAAGAAGCCTCGATGTGGTGTAC-3′) and reverse primers (5′-CTTCTGCCCAGTTAATGCATAGC-3′) in a reaction volume of 25 µl. Primers were designed using Primer Express software (Applied Biosystems). Real-time quantification was performed by measuring the increase in fluorescence caused by the binding of SYBR Green dye to double-stranded DNA at the end of each amplification cycle. Relative expression was determined by using the ΔΔCt (threshold cycle) method for normalized samples (ΔCt) relative to a calibrator sample, according to the manufacturer's protocol. Each PCR run included a no-template control and a sample without reverse transcriptase. All measurements were performed in triplicate.

### Statistical analysis

To test whether the genotypes were in Hardy-Weinberg equilibrium, we compared the observed genotype frequencies with their expected frequencies at equilibrium, based on the Chi2 test. Odds ratios (Ors) and p values adjusted for birth weight and ethnic origin, obtained by logistic regression, were calculated to test the association between the genotypes and BPD. Differences between treatment groups in animal studies were evaluated with the Kruskal-Wallis non-parametric test for multiple group comparisons, and the Mann-Whitney U test for two-group comparisons.

## Results

### Study population

All infants were inborn, and received prophylactic surfactant in the delivery room. Of the 284 neonates initially enrolled, 21 died before 36 weeks PMA and were not included in the analysis. Death was mainly due to severe neurological or infectious complications. Among survivors, 48% had histologically defined chorioamnionitis, 8% had bacteriologically proven maternofetal infection, 76% received medical treatment for persistent ductus arteriosus, 16% had bacteriologically proven postnatal sepsis, and only one had air leak. Ninety-two percent of mothers had received partial or complete steroid treatment before delivery. Forty-five (17%) of the other 263 infants still needed oxygen supplementation at 36 weeks PMA and were thus diagnosed with BPD. As expected, the risk for BPD was significantly associated with birthweight, persistent ductus arteriosus, postnatal sepsis, and chorioamnionitis in univariate analysis (Table 2). Birthweight was the only significant risk factor for BPD in multivariate analysis (OR (95%CI) = 0.996 (0.993–0.998); p = 0.0017), and was therefore used as adjustment factor in genetic analysis.

**Table 2:** Univariate analysis of perinatal factors potentially influencing the risk of BPD.

Variable		Infants with BPD/total (%)	OR	95% CI	*P*
Sex	Female	20/132 (15)	1		
	Male	25/131 (19)	1.321	0.693–2.518	0.398
Ethnicity*	European	19/97 (20)	1		
	North Africa	3/23 (13)	0.616	0.166–2.289	0.469
	Sub-Saharan Africa	15/103 (15)	0.700	0.333–1.470	0.346
	Other	8/40 (20)	1.026	0.408–2.583	0.956
Birth weight (100 g)			0.585	0.453–0.755	<0.0001
Gestational age (wk)			0.804	0.590–1.096	0.167
Maternofetal infection	No	41/237 (17)	1		
	Yes	2/21 (10)	0.503	0.113–2.245	0.368
Persistent ductus	No	4/63 (6)	1		
arteriosus§	Yes	39/195 (20)	3.687	1.263–10.770	0.017
Surgical treatment for	No	32/219 (15)	1		
ductus arteriosus	Yes	11/39 (28)	2.296	1.040–5.068	0.040
Chorioamnionitis	No	22/105 (21)	1		
	Yes	7/99 (7)	0.287	0.117–0.707	0.007
Postnatal sepsis	No	32/218 (15)	1		
	Yes	12/41 (29)	2.405	1.113–5.196	0.025
Antenatal steroid	No	3/21 (14)	1		
therapy	Partial	16/76 (21)	1.600	0.419–6.117	0.492
	Complete	25/163 (15)	1.087	0.298–3.966	0.899

* Ethnic group was based on the common origin of both parents; newborns whose parents had different ethnic origins were classified as “other” ;

§ Need for medical treatment

### Genotyping

The observed genotype frequencies did not deviate from Hardy-Weinberg equilibrium. The genotype distribution of 7 of the 9 studied SNPs was significantly influenced by ethnic origin. Table 3 shows the frequency of BPD in infants with different genotypes. After adjustment for birth weight and ethnic origin, the TT genotype of MMP16 C/T and the GG genotype of MMP16 A/G were found to be associated with a significantly lower risk of BPD (Table 3). The T allele frequency of the MMP16 C/T polymorphism was 50% in infants without DBP, as compared with only 34% in infants with BPD (p = 0.01). Similarly, the G allele frequency of the MMP16 A/G polymorphism was found to be significantly higher in infants without BPD than in those with BPD: 37% versus 23%, respectively (p<0.03). Twin cases did not significantly change results. Among survivors, 17 dizygotic twin pairs, 1 monozygotic twin pair, and 4 triplet pairs were present. Adjusted results for multiple births still identified TT and GG genotypes as significantly associated to a lower risk of BPD (p<0.02 and <0.05, respectively).

**Table 3:** SNP genotypes and frequency of BPD in infants; odds ratio and P values for the association between genotype and BPD, after adjustment for birth weight and ethnic origin, using logistic regression.

Gene and position of SNP	Genotype	Infants with BPD/total (%)	Adjusted OR	95% CI	P
MMP2	CC	30/162 (19)	-		
−1306	CT	11/44 (25)	1.123	0.446–2.828	0.806
	TT	0/4 (0)	NC		
MMP14	GG	25/141 (18)			
−129	GT	12/82 (15)	0.877	0.395–1.946	0.747
	TT	2/21 (10)	0.499	0.103–2.423	0.388
MMP14	TT	30/179 (17)	-		
+256	TC	9/59 (15)	0.667	0.280–1.587	0.359
	CC	0/7 (0)	NC		
MMP14	CC	31/178 (17)			
+6762	CG	9/67 (13)	0.905	0.377–2.175	0.823
	GG	2/10 (20)	1.395	0.249–7.822	0.705
MMP14	GG	34/203 (17)			
+6802	GA	8/48 (17)	0.823	0.328–2.062	0.678
	AA	0/4 (0)	NC		
MMP14	CC	29/163 (18)			
+6926	CT	10/78 (13)	0.684	0.290–1.613	0.386
	TT	3/14 (21)	1.109	0.268–4.579	0.886
MMP14	TT	21/131 (16)			
+7131	TC	17/96 (18)	1.239	0.581–2.644	0.579
	CC	4/28 (14)	1.327	0.387–4.553	0.653
MMP16	AA	25/113 (22)			
+39811	AG	16/100 (16)	0.668	0.320–1.395	0.282
	GG	2/35 (6)	0.208	0.044–0.978	0.047
MMP16	CC	19/76 (25)			
+43827	CT	19/112 (17)	0.579	0.270–1.241	0.160
	TT	5/60 (8)	0.244	0.080–0.744	0.013

NC = not calculated

Twenty-one infants died before 36 weeks of post-menstrual age. Among clinical factors significantly associated to the development of BPD in our population (Table 2), only birthweight (747±36 g) was found to significantly differ in deceaded infants from alive infants without BPD. None of SNPs were associated to death. When evaluating the composite outcome of death or BPD by 36 weeks of post-menstrual age, adjusted OR associated to GG and TT genotypes were 0.406 (0.126–1.310; p = 0.1315) and 0.447 (0.184–1.085 ; p = 0.0753), respectively.

Power of our study results from the effect of SNP on disease (odds ratio), the sample size (n) and the type I error risk (alpha). When considering a multiplicative model for the allele associated with BPD, and the 45 children with BPD among 263 preterm infants recruited in this study, for alpha = 5%, the power of the one-sided test to detect an association between BPD and an allele is equal to 99% with a C/T frequency of 0.50 and an odds ratio of 3.8 or is equal to 98% with a G/A frequency of 0.37 and an odds ratio of 3.3. If we take into account that the two SNPs found positively associated to BPD were tested among 9 candidates, group sample sizes of 45 and 218 achieve 82% power for each gene to detect a true allelic difference of at least 0.15 and with a false discovery rate of 0.05.

### MMP2 level in tracheal aspirates

**Figure 1:**
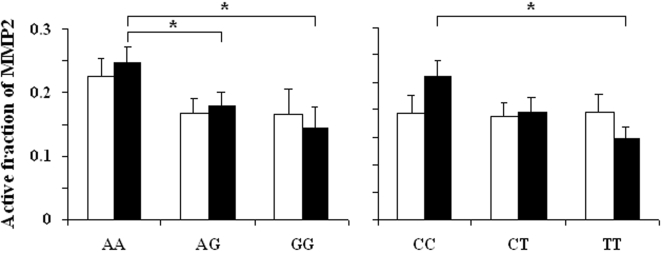
Association between MMP16 polymorphisms (A: rs2664349 A/G; B: rs2664352 C/T) and the size of the activated fraction of MMP2 measured in tracheal aspirates. Aspirates were collected immediately after birth (open bars), and between 1 and 3 days of life (closed bars). * p<0.05 in post-hoc analysis.

The mean MMP2 protein content and activity did not differ significantly on day 0 between infants who subsequently developed BPD and those who did not (Table 4). However, second samples had significantly higher MMP2 contents and higher levels of activated MMP2 in BPD infants. The pattern of change in the activated MMP2 fraction differed significantly according to BPD status, with lower levels at birth in BDP infants, followed by a steep increase during the first days of life (p = 0.0001, repeated measures ANOVA). A significant association was found between polymorphisms in the MMP16 gene and the activated MMP2 fraction in second samples, but not in first samples (day 0) (Figure 1). No significant association was found between the MMP2 or MMP14 genotypes and MMP2 activity in tracheal aspirates (data not shown).

**Table 4:** MMP2 ELISA values and zymographic analysis of MMP2 activity in tracheal effluents of premature newborns.

	BPD −	BPD +	p
**Initial sample (H0)**	n = 126	n = 22	
MMP2 content (ng.mL-1)	1.36±0.06	1.42±0.08	NS
Total activity (AU.mL-1)	2.88±0.06	3.03±0.09	NS
Activated form (AU.mL-1)	1.96±0.07	1.84±0.12	NS
Activated form (% of total)	19.7±1.5	9.8±2.0	0.009
**Second sample (day 1 to 3)**	n = 89	n = 16	
MMP2 content (ng.mL-1)	1.29±0.04	1.51±0.09	0.034
Total activity (AU.mL-1)	2.72±0.06	3.00±0.08	0.054
Activated form (AU.mL-1)	1.82±0.07	2.29±0.09	0.009
Activated form (% of total)	19.0±1.6	26.0±4.3	NS

Infants were ascribed to one of two subgroups according to their BPD phenotype. Values are means±SEM and were log transformed to respect a normal distribution.

### MMP16 protein content in human samples

**Figure 2:**
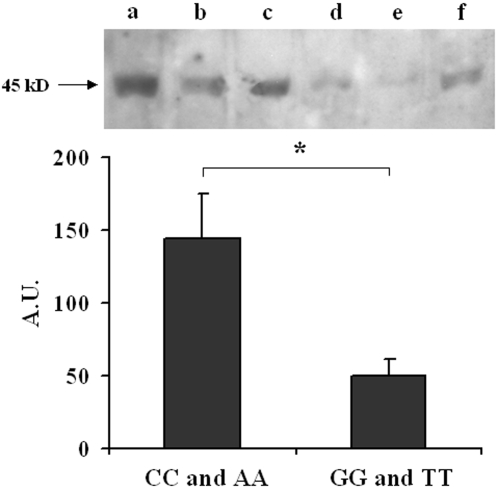
MMP16 immunoblots of tracheal aspirate supernatants from infants with the MMP16 AA-CC (lanes a–c) and GG-TT (lanes d–f) genotypes. Upper: a 45-kDa band was clearly identified in AA-CC infants but was barely visible in GG-TT infants. Lower: densitometric analysis (arbitrary units [A.U.]) in 9 AA-CC infants and 8 GG-TT infants, showing a significant difference in the MMP16 level (Mann Whitney p<0.02).

Given this association between the MMP16 genotype and MMP2 activation, we used immunoblot to determine MMP16 protein content in supernatants of tracheal aspirates from infants with homogenous genotypes, namely GG and TT (n = 8) or CC and AA (n = 9). A single 45-kD band was observed in every case. Tracheal aspirates from infants with the GG-TT genotype contained three times less MMP16 protein than those from infants with the CC-AA genotype (Figure 2).

**Figure 3:**
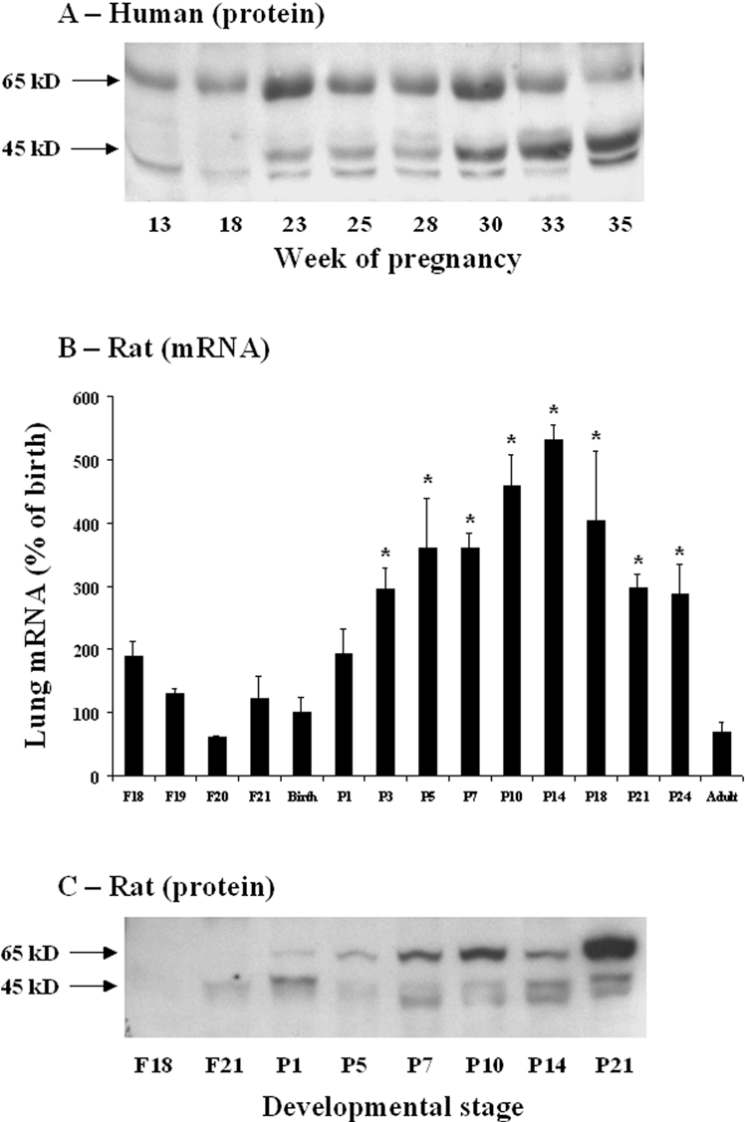
Developmental pattern of MMP16 gene and protein expression in control lungs. A. Immunoblot of lung homogenates from human fetuses without lung disease, at 11 to 36 weeks of gestation. B. Changes in the MMP16 mRNA expression level in rat whole lung tissue. Expression was quantified by real-time PCR from fetal life (canalicular stage of development) to adulthood, in 3 to 5 individual lung samples per stage. The birth level was arbitrarily given a value of 100. Values are means±SEM. * p<0.05 versus birth level. C. Immunoblot of lung homogenates from control rats, from fetal life to adulthood.

MMP16 protein expression was also determined in lung tissue from eight deceased fetuses without lung disease, sampled between 13 and 35 weeks of pregnancy (Figure 3A). Three bands were observed, at 65 kD, 45 kD, and ∼35 kD. While expression of the 65-kD band remained stable during fetal life, expression of the lower-molecular-weight species rose sharply from the 30th week of pregnancy.

### MMP16 expression in rat lung

#### Expression profile of MMP16 mRNA and protein from fetal life to adulthood

The MMP16 mRNA level was evaluated in developing rat lung from fetal day 18 (canalicular stage) to adulthood. Gene expression changed little until postnatal day 1, then increased gradually to about five times the fetal level on day 14, remained elevated until day 24, and returned to an extremely low level (lower than before birth) in adulthood (Figure 3B). As in human tissue, immunoblotting showed three bands, with molecular weights of 65 kD, 45 kD, and ∼35 kD. Expression of all the bands increased from postnatal day 5 to postnatal day 21, but the strongest increase was observed for the 65 kD band (Figure 3C). Confirmatory experiments were performed with quantification of protein expression in 4 individual samples at days 1, 7, and 21. A significant increase was found for the 65 kD protein (Table 5). The 45 kD form increased slightly from day 1 to day 21, but the difference was not significant.

**Table 5:** Densitometric analysis (arbitrary units±SEM) of immunoblots of lung homogenates from control rats at three postnatal ages: day 1, day 7, and day 21 (n = 4 at each stage).

	Day 1	Day 7	Day 21
65 kD	726±223	1277±314	2308±385*
45 kD	1374±282	757±193	1802±83

Ponceau S stain was used as loader control. Normalization of immunoblots was achieved through the run of a common sample.

* p<0.05 as compared with day 1.

#### Effects of hyperoxia and dexamethasone on MMP16 gene expression in postnatal rat lung

**Figure 4:**
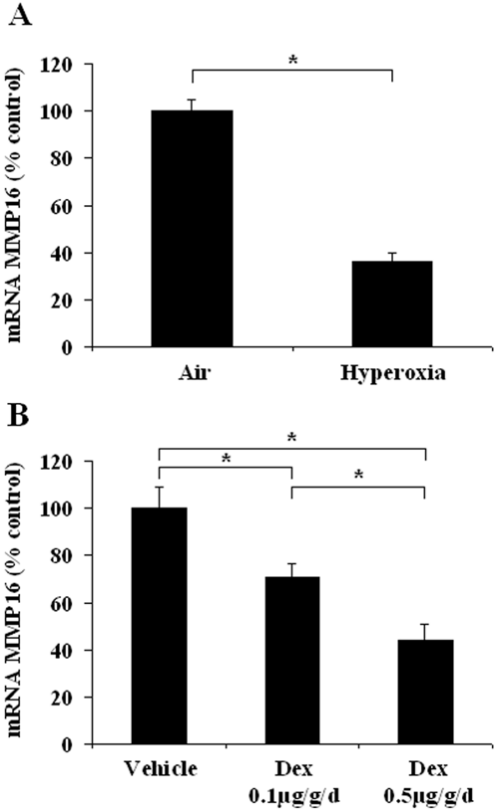
Changes in lung MMP16 mRNA expression in newborn rats exposed to insults associated with alveolar developmental arrest. A: Exposure to hyperoxia (O_2_>95%) from birth to day 6; B: Daily administration of dexamethasone from birth to day 5 (two doses). Results are expressed as a percentage of the control value. * p<0.05 in two-group comparisons.

In newborn rats exposed to hyperoxia for 7 days, the MMP16 gene expression level (Figure 4A) was about 60% lower than in controls. Similarly, dexamethasone caused a dose-dependent fall in the MMP16 mRNA level. MMP16 gene expression fell by 30% and >50%, respectively, with the 0.1 and 0.5 µg/kg/d dexamethasone dosages (Figure 4B). Protein changes, evaluated by immunoblotting, were concordant with RNA changes. Densitometry results, expressed as a percentage of the control value, showed that the 65 kD form decreased to 28±5% (n = 4 ; p<0.03) and to 48±8% (n = 4 ; p<0.03), after hyperoxia or 0.5 µg/kg/d dexamethasone, respectively. The changes in the 45 kD form (48±15%, and 176±32% of control values, respectively) were not found to be significant.

## Discussion

Genetic susceptibility to BPD was demonstrated in a recent study of monozygotic and dizygotic twins [12]. After controlling for covariates, genetic factors were shown to account for 53% of the variance in the risk of BPD [12]. Genes encoding multifunctional proteins in the distal lung are prime candidates for determining susceptibility to BPD.

We sought links between BPD and SNPs in three MMP genes. We found a significant association between two SNPs in the MMP16 gene and BPD outcome, even after adjustment for birth weight and ethnic origin. The MMP16 genotype also influenced the size of the activated MMP2 fraction in the infants' tracheal aspirates. In addition, MMP16 gene expression was high during normal newborn rat lung alveolarization, and was markedly reduced by insults that arrested alveolarization.

Heterogeneous management of preterm infants hampers genetic analyses. Indeed, the frequency of BPD varies widely among neonatal intensive care units, underlining the role of environmental factors. The prospective nature of our study helped to ensure that newborns who did and did not subsequently develop BPD received similar initial management. The rate of BPD was low (17%), reflecting good control of external insults. Furthermore, oxygen dependency at a postmenstrual age of 36 weeks was diagnosed with a validated test [15].

No association was found between the risk of BPD and SNPs in the MMP2 or MMP14 gene. The SNPs we selected have previously been linked to human respiratory diseases, including COPD [14], and lung cancer [19], [20]. The proteinases produced by these two genes play an important role in the control of distal lung development [4], [7]–[9]. MMP2 has also been implicated in the pathogenesis of BPD [5], [6]. In the present study, MMP2 content and activity were measured in tracheal effluents. The main finding was the association between the risk of BPD and the proportion of the activated fraction of MMP2. Relative to infants who did not develop BPD, infants who developed BPD had a significantly smaller fraction of active MMP2 at birth, but a larger fraction at the second sampling time. These results support our previous findings [5]. They are also in keeping with the early increase in the active MMP2 fraction previously observed in the lungs of newborn rats exposed to hyperoxia [21]. However, contrary to our previous findings, total MMP2 activity did not differ on day 0 between neonates who subsequently developed BPD and those who did not in this study. Differences in the study populations might account for this apparent discrepancy. In the present study the neonates were more premature, were all sampled within minutes after birth, and all received prophylactic surfactant administration. No association was found between the MMP2 and MMP14 genotypes and MMP2 levels in tracheal aspirates. By contrast, the MMP16 genotype was associated both with the risk of BPD and with the level of MMP2 activity in tracheal aspirates. In keeping with our observation that BPD was associated with an elevated active fraction of MMP2, the MMP16 genotypes associated with a lower risk of BPD, i.e. GG and TT, were also associated with lower active fractions of MMP2.

MMP16, also called MT3-MMP, is very similar to MMP14 in both structure and function. It is present in membranes as an active 65-kD form, and has recently been shown to be shed in a 32- to 35-kD soluble form that retains its ability to activate MMP2 [22]. A 45-kD soluble form of MMP16 has also been suggested to be formed by alternative splicing of mRNA [11], the splicing point potentially lying in the hemopexin-like domain. It was recently suggested that the two forms of this protein could play distinct roles during embryogenesis. Indeed, Xenopus laevis embryos injected with full-length MMP16 mRNA showed no significant changes in the expression levels of the tissue-specific genes encoding endodermin, chordin and muscle actin, whereas mRNA for the soluble form of MMP16 reduced the expression of all three marker genes. In addition, while full-length tethered MMP16 failed to alter gelatinase activity, a 50% increase occurred after injection of the soluble form [23]. MMP16 was recently shown to act as a major collagenolytic enzyme in bone and cartilage during mammalian embryogenesis, with MMP16-deficient mice displaying delayed skeletal growth [24]. Although no genotype-related abnormality is reported in lungs of adult mutant mice in the international database resource for the laboratory mouse (http://www.informatics.jax.org/external/ko/deltagen/1241.html), lung development was not extensively studied in these mice. MMP16 is known to be expressed in the human lung [10]. Its role is largely obscure, but it has been implicated in disease processes such as idiopathic pulmonary fibrosis [25]. Our study is therefore the first to provide strong evidence of MMP16 participation in normal and abnormal lung development, in both newborn rats and premature infants. In newborn rats, lung MMP16 gene expression is low during the canalicular and saccular stages, and increases significantly very close to the beginning of alveolarization, i.e. postnatal day 4 [26]. Then, lung MMP16 gene expression peaks on day 14 and remains elevated until day 24 of life, before falling to very low levels in adulthood. MMP16 protein expression in rat lung ran parallel to MMP16 gene expression, with a gradual increase in both the presumed transmembrane and soluble forms during alveolarization, and strong expression on day 21. The results we obtained with homogenates of normal human fetus lungs sampled at various stages were concordant with the results we obtained in rats. We observed a clear increase in the expression of the presumed soluble form as early as 30 weeks of human pregnancy, i.e. just before the beginning of alveolarization [26]. Although these observations were gained from tissues collected postmortem, these samples have previously been proven suitable for evidencing developmental changes as well as differences between normal and diseased lungs [17], [27]. We found that the pulmonary MMP16 expression profile differed from that of MMP14 in lung fibroblasts from newborn rats. MMP14 gene expression increased at the time of peak alveolarization but rapidly returned to the basal level as early as 16 days of life [7]. Although MMP16 is closely related to MMP14 in terms of its molecular structure and tissue expression pattern [10], [24], differences in the expression profiles suggest that MMP16 is not merely functionally redundant to MMP14. Alveolarization takes place between days 4 and 21 in rats and comprises two successive steps, namely secondary alveolar septation from day 4 to day 14, and alveolar wall thinning with fusion of the initially double capillary layer into a single, central microvascular network from day 14 to 21 [26]. The different expression profiles of MMP14 and MMP16 suggest primary involvement of the former in the first step and of the latter in the whole process, including the second step.

The functional domains of MMP16 and MMP14 are not interchangeable for pro-MMP2 activation and collagenolysis, despite their similarities [28]. It was also shown recently that the collagenolytic activity of MMP16 is complementary to that of MMP14 in mice lacking both enzymes [24]. Further evidence supporting the role of MMP16 as a significant controller of alveolar growth/maturation comes from its down-regulation in models of arrested of lung development. We found that hyperoxia and dexamethasone, both of which impair alveolarization, strongly reduced lung MMP16 gene expression in newborn rats. We also observed a significant association between two polymorphisms in the MMP16 gene and the risk of BPD in highly premature infants. Despite strong evidence of genetic susceptibility to BPD [12], only polymorphisms in the genes for glutathione-S-transferase-P1 [29], surfactant protein-B [30], and TNF-a [31] have been identified as possible genetic determinants. The functionality of the two SNPs that we studied in the MMP16 gene remains to be determined. They were chosen because they are tags for a haplotype in a gene region that codes for the hemopexin-like domain and that might be alternatively spliced [11]. The association we found between the infants' genotypes and both the MMP16 protein level and the size of the activated MMP2 fraction in tracheal aspirates strongly suggests that these polymorphisms have functional consequences, although no definitive proof is given that the changes in MMP16 are causative of reduced MMP2 activation.. The premature infants had a gestational age lower than 28 weeks, corresponding to the early saccular stage of lung development. We found that expression of the 45-kD soluble form of MM16 was low in human fetal lung tissue. The TT and GG genotypes were associated with a 3-fold lower 45-kD MMP16 protein content in tracheal aspirates collected within 3 days after birth, and with a smaller MMP2 active fraction, in keeping with experimental results linking expression of the soluble MMP16 form to the level of gelatinase activity [23]. These genotypes were also found to be associated with a significantly lower risk of BPD, suggesting that maintenance of the physiologically low expression of the 45-kD soluble MMP16 isoform at this stage of lung development protects against BPD. Apparent discrepancies between changes in MMP16 expression in BPD infants and changes in animal models of arrested alveolarization may be due to differences in time frame. Animal experiments are models of arrested alveolar septation, a stage associated to a strong physiological increase in MMP16 expression. Thus, in both BPD infants and animal models, altered distal lung development is associated to opposite changes in MMP16 expression as those normally observed in the corresponding lung development stage. It is also noteworthy that changes in protein expression in animal models were found significant only for the tissular 65 kD form. For evident reasons, we were not able to evaluate this tissular form in human prematures. Changes in balance between tissular and soluble forms may also contribute to lung growth disorders.

In conclusion, we identify MMP16 as a possible new regulator of lung alveolar development and provide evidence that MMP16 polymorphisms are associated with protection from bronchopulmonary dysplasia in highly premature infants.
